# Organ Reconditioning and Machine Perfusion in Transplantation

**DOI:** 10.3389/ti.2023.11100

**Published:** 2023-01-12

**Authors:** Maria Irene Bellini, Eliano Bonaccorsi Riani, Emmanouil Giorgakis, Maria E. Kaisar, Damiano Patrono, Annemarie Weissenbacher

**Affiliations:** ^1^ Department of Surgery, Sapienza University of Rome, Rome, Italy; ^2^ Abdominal Transplant Unit, Cliniques Universitaires Saint-Luc, Université Catholique de Louvain, Brussels, Belgium; ^3^ Pôle de Chirurgie Expérimentale et Transplantation - Institute de Recherche Expérimentale et Clinique, Université Catholique de Louvain, Brussels, Belgium; ^4^ Department of Surgery, University of Arkansas for Medical Sciences, Little Rock, AR, United States; ^5^ Division of Solid Organ Transplantation, UAMS, Little Rock, AR, United States; ^6^ Nuffield Department of Surgical Sciences, Medical Sciences Division, University of Oxford, Oxford, United Kingdom; ^7^ Molinette Hospital, Turin, Italy; ^8^ Department of Visceral, Transplant and Thoracic Surgery, Center of Operative Medicine, Medical University of Innsbruck, Innsbruck, Austria

Society’s self-sufficiency in terms of organ replacement is still far away from being achieved, given the large disparity between transplant demand and donor organ availability. In the attempt to reduce this discrepancy and expand the donor pool, the transplant community has progressively increased the utilisation of extended-criteria donors (ECD) such as older donors, donors with comorbidities and donation after circulatory death (DCD). The main challenge preventing a wider utilisation of ECD and DCD allografts is the higher susceptibility to the ischemia-reperfusion injury (IRI) (1), an unavoidable part of the transplantation process. For this reason, in the last decades, there has been an exponential development on organ reconditioning strategies, in order to enable graft resuscitation and viability assessment prior to implantation (2).

*Ex-vivo* perfusion techniques are usually classified according to the perfusate temperature, hypothermic (<10°C) or normothermic (37°C), with roller or centrifugal pumps used to generate pressure-controlled pulsatile or continuous flow within the organ, *via* connection to the renal inflow (artery) and outflow (vein) (3). Given the variety of combinations of different parameters and settings (temperature, oxygen, nutrient and/or drug delivery, *in situ*/*ex-situ*), machine perfusion (MP) is considered a promising way to expand the criteria of transplantation by optimising its preservation modalities, potential of organ viability assessment and potentially decreasing the rate and gravity of postoperative complications (4).

As the commitment of Transplant International is to be the premier journal publishing the key basic science and clinical developments in organ replacement medicine (5), we aimed to develop a special issue reflecting the current state of the art on MP in transplantation. This topic collection is dedicated to Professor Paolo Muiesan (6), as a tribute to his career accomplishments in the field of dynamic organ preservation.

In the present issue, we touched upon the whole spectrum of thoracic and abdominal transplant surgery: from heart preservation (Qin et al.), whose valuable contribution was key to allow the first cardiac xenograft to happen (7), to normothermic lung perfusion, enriched by precise measurement of exhaled endogenous CO (Brenckmann et al.), to provide an additional early marker in lung grafts evaluation, moving toward the current trends in preservation solutions for pancreas (Ferrer-Fàbrega et al.), for normothermic kidney and hypothermic combined kidney-liver technologies (Fard et al.; Chang et al.), and describing the first proof-of-concept pilot study for liver viability assessment, *via* hyperspectral imaging (Fodor et al.).

Indeed, viability assessment stays at the core of the present topic collection, in fact the prediction of future organ functionality before transplantation remains the main challenge to pursue on the decision to implant a non-standard criteria graft. This leads roughly to a 20% liver and kidney discard, based on the transplant provider’s judgement, rather than following objectively identifiable scores, which are currently not available for most of the areas discussed. Thus, the development of MP platforms, particularly under normothermia, where more physiological conditions could be reproduced, embraces the potential to overcome this subjective evaluation (Verstraeten and Jochmans). There are in fact novel biomarkers and therapeutic strategies currently under investigation, mainly including pH and electrolytes (Dingfelder et al.), that promise to acquire reliability in view of their association with outcomes after implantation.

In more detail, normothermic MP of liver grafts builds on improvements in preoperative management, surgical technique (8), and postoperative care that successfully allowed to increase the safe use of non-standard criteria grafts, through objective assessment of both hepatocyte and cholangiocyte function (Hann et al.). Machine preservation technology is proved to minimize ischemic biliary complications, too (9, Hunt et al.). It has been recently published the clinical success of an extended time window of up to 10 days of storage prior to implantation, unveiling new horizons in research and clinical utility, namely to convert an urgent and highly demanding surgery into an elective procedure (10).

Dynamic organ perfusion technology represents a significant advancement in graft preservation techniques and the transplant community must seek to continue to incorporate future developments to ensure the benefits of organ transplantation are maximized. Yet, there are also downsides to be considered, specifically to increase MP cost-effectiveness. In the present special issue, a hospital-based health technology assessment of MP in adult liver transplantation using standard cold storage as a comparator, and within the perspective of a national health system-based hospital practice and disease-related group reimbursement policy was presented (De Simone and Ghinolfi). Results showed that the choice of the most appropriate costing model and resource-use items are crucial and require broad consensus across the healthcare professionals involved in transplant programs. The decision on which types of cost to include depends on several key factors, such as the perspective to be adopted, the form of economic evaluation (e.g., cost-effectiveness versus cost utility versus cost opportunity), the quantitative importance of the type of cost, along the entire transplant continuum, whether the cost can be attributed to the intervention and the time horizon of the economic evaluation (perioperative versus early-term versus long-term versus life-long). Furthermore, there is also a non-uniform MP practice among experts in the field highlighting the need for more focused research (11).

To this regard, it is worth mentioning that some techniques are not competitive, but rather complementary, such as abdominal normothermic regional perfusion preceding dual hypothermic oxygenated MP for controlled DCD liver transplantation. As highlighted in one of the included manuscripts (Patrono et al.), this combination allowed to achieve recipient outcomes comparable to livers retrieved from donors after brain death.

The encouraging results from DCD organs utilised after normothermic regional perfusion are also shown by the Edinburgh experience herein presented (Hunt et al.). Their implementation model overcame the barriers associated with the adoption of a new technique, leading to standard clinical practice *via* an iterative process of refinement, training, staffing and operative techniques. Using this approach, the authors achieved a four-fold increase in trained surgical staff and a six-fold increase in competent senior organ preservation practitioners in 12 months. The 61 DCD liver transplants undertaken exhibited no primary non-function or ischaemic cholangiopathy with up to 8 years of follow-up.

In conclusion, we hope this special issue will become a state of the art collection on the use of dynamic organ preservation. The possibility to inspect organ function and to limit the ischemic injury, its consequences in terms of primary or delayed graft function (Hunter et al.) and early dysfunction are key for the future of transplantation, to improve graft and patient survivals.
